# Sex differences in PTSD speech biomarkers assessed by virtual agent-induced conversations

**DOI:** 10.3389/fpsyg.2025.1509206

**Published:** 2025-04-28

**Authors:** Felix Menne, Louisa Schwed, Felix Dörr, Nicklas Linz, Johannes Tröger, Alexandra König

**Affiliations:** ^1^ki:elements GmbH, Saarbrücken, Germany; ^2^Cobtek (Cognition-Behaviour-Technology) Lab, University Côte d’Azur, Nice, France; ^3^Centre Hospitalier et Universitaire, Clinique Gériatrique du Cerveau et du Mouvement, Centre Mémoire de Ressources et de Recherche, Université Côte d'Azur, Nice, France

**Keywords:** PTSD, speech, speech biomarkers, sex differences, gender differences, automated speech analysis

## Abstract

**Introduction:**

Women face a substantially elevated risk of developing PTSD compared to men. With the emergence of automated digital biomarkers for assessing complex psychiatric disorders, it becomes imperative to take into account possible sex differences.

**Objectives:**

Our objective was to explore sex-related speech differences in individuals with PTSD.

**Methods:**

We utilized data from the DAIC-WOZ dataset, consisting of dialogs between participants with PTSD (*n* = 31) and a virtual avatar. Throughout these dialogs, the avatar utilized diverse prompts to maintain a conversation. Features were extracted from the transcripts, and acoustic features were obtained from the recorded audio files. Group comparisons, correlations, and linear models were calculated to assess sex-related differences in these features between male and female individuals with PTSD.

**Results:**

Group comparisons yielded significant differences between male and female patients in acoustic features such as the F2 frequency Standard Deviation (higher in males) and Harmonics to Noise Ratio (lower in males). Correlations revealed that Loudness Standard Deviation was significantly associated with PCL-C scores in males, but not in females. Additionally, we found interaction effects for linguistic and temporal features such as verb phrase usage, adposition rate, mean utterance duration, and speech ratio, with males showing positive associations and females showing inverse associations.

**Conclusion:**

Sex-related variations in the expression of PTSD severity through speech suggest contrasting effects in acoustic and linguistic features. These results underscore the importance of considering sex-specific expressions of behavioral symptoms in developing digital speech biomarkers for diagnostic and monitoring purposes in PTSD.

## 1 Introduction

Posttraumatic stress disorder (PTSD) is a frequent psychiatric indication with lifetime prevalence numbers up to 26.9% of the population (Schein et al., 2021). PTSD arises from exposure to a traumatic life event and is characterized by four discernible symptom clusters: (1) re-experiencing of the traumatic event, manifested through phenomena like dreams, flashbacks, and intrusive, distressing thoughts; (2) avoidance and numbing, marked by behaviors such as avoiding trauma reminders and experiencing emotional numbing; (3) hyperarousal, characterized by difficulties in sleeping and concentrating, irritability, and hypervigilance; and (4) negative alterations in cognitions and mood, such as the inability to remember an important aspect of the traumatic event or negative beliefs or expectations about oneself, others, or the world (American Psychiatric Association and DSM-5 Task Force, 2013).

Women face a two to three times higher risk of developing PTSD than men after traumatic experiences (Olff, 2017), especially at younger ages (Hodes and Epperson, 2019). This increased risk is linked to factors such as the type of trauma experienced, younger age at the time of trauma, a stronger perception of threat, and higher levels of peritraumatic dissociation (Olff et al., 2007; Irish et al., 2011). However, despite controlling for disparities in trauma types, studies indicate persistent sex differences in incidence and prevalence (Blanco et al., 2018; Christiansen and Berke, 2020). Hormonal variances, particularly the roles of estradiol and progesterone in emotional memory consolidation, along with sex-specific genetic and epigenetic factors, may contribute to the increased susceptibility of women to PTSD following traumatic events (Glover et al., 2015; Ramikie and Ressler, 2018; Ney et al., 2019; Christiansen and Berke, 2020).

Evidence also suggests disparities in the experience and perception of post-traumatic stress symptoms (PTSS) between women and men. Studies indicate that women tend to report more acute PTSS than men (Elklit, 2002; Bryant and Harvey, 2003; Hetzel-Riggin and Roby, 2013), potentially contributing to a higher likelihood of developing PTSD. Further investigations reveal sex-specific differences in symptom expression, with women displaying elevated levels of re-experiencing, avoidance, emotional numbness, and hyperarousal compared to men (Hourani et al., 2015; Farhood et al., 2018). Additionally, a study by Hetzel-Riggin and Roby (2013) demonstrated that women generally report more PTSS than men.

Many individuals with PTSD go undiagnosed for various reasons. Objective measures of symptom severity are lacking, relying predominantly on subjective assessments through questionnaires, interview protocols, and scales [e.g., Clinician Administered PTSD Scale, CAPS (Weathers et al., 2018); the PTSD Symptom Scale, (Foa et al., 2016)]. Notably, sex differences in PTSD symptomatology may contribute to discrepancies in assessments, as men and women may exhibit different symptom expressions and face varied stigma when discussing traumatic experiences (Tolin and Foa, 2006). The heterogeneous phenomenology of PTSD, coupled with overlaps with other psychiatric conditions like depression, poses challenges for accurate symptom classification (Meltzer et al., 2012). Reluctance to share traumatic experiences due to stigma, guilt, or shame, further hinders accurate diagnosis and timely treatment (Lee et al., 2001; Silvestrini and Chen, 2023). Consequently, only an estimated 35% of PTSD patients seek treatment, often delayed (Nobles et al., 2016) which points to a need for better and earlier detection of PTSD symptoms.

In recent years, a growing number of sensors and digital biomarkers have emerged to objectively capture behavioral or biological information for psychiatric disorders (Jacobson et al., 2019; Schultebraucks et al., 2020; Menne et al., 2024). These tools have proven useful not only in PTSD research but also in other conditions, such as Alzheimer’s Disease (de la Fuente Garcia et al., 2020) and cognitive and thought disorders (Voleti et al., 2020), where similar neural and behavioral disruptions are observed. Among these, automated speech analysis presents significant opportunities for studying disease-related characteristics (Malgaroli and Schultebraucks, 2020). Psychiatric symptoms often manifest in speech and language, making it essential for clinical assessments to consider patients’ speech patterns, including speed, coherence, and content. Advances in computer linguistics, natural language processing, and speech recognition facilitate the use of automatic speech analysis as an objective clinical measurement of psychiatric symptoms (König et al., 2022, 2023; Menne et al., 2024).

Concerning PTSD, research has explored the diagnostic potential of speech biomarkers, revealing that PTSD is associated with altered word choices on a lexical level, such as the usage of more emotional words, pronouns and adjectives (Pennebaker et al., 2003; Jaeger et al., 2014; Papini et al., 2015). Also, it has been demonstrated that the increased severity of PTSD symptom clusters is associated with differing linguistic characteristics. For instance, PTSD individuals with increased severity of reexperiencing symptoms less often use words related to death and dying (Papini et al., 2015). Additionally, on an acoustic level, it is characterized by more monotonous, slower, and flatter speech (Marmar et al., 2019; Low et al., 2020). However, research on speech differences in PTSD has primarily controlled for sex by creating sex-matched samples, with little attention given to examining differences in speech features between the sexes. The literature on sex differences in speech within the field of psychiatry is limited. Hönig et al. (2014) conducted a study on the automatic modeling of depressed speech and identified trends indicating variations in spectral and prosodic features between males and females. Cummins et al. (2017) also observed that accounting for sex differences can improve speech-based depression detection, particularly through the influence of vowel-level formant features. To the best of our knowledge, there are no comparable studies specifically addressing sex effects on speech in PTSD. Pursuing the approach of precision psychiatry (Bzdok and Meyer-Lindenberg, 2018) and with automated digital biomarkers emerging to aid in the characterization of complex psychiatric disorders (Jacobson et al., 2019; Chen et al., 2022), it is imperative to account for potential sex differences in these biomarkers, ensuring accurate diagnosis, and effective treatment customization (Cirillo et al., 2020). In a draft guidance, the Food and Drug Administration (FDA) recently emphasized the importance of analyzing sex-specific data in clinical trials to better understand the differential effects of medical products on male and female populations, ensuring that treatment benefits and risks are accurately assessed for both sexes (Food and Drug Administration, 2025).

This exploratory study investigates speech differences between sexes in a sample of individuals with PTSD. Beyond apparent sex differences such as pitch, additional distinctions in word count, intelligibility, and prosody between sexes have been identified in non-diseased individuals (Whiteside, 1996; Besson et al., 2002; Leaper and Ayres, 2007). Given the sex differences in the expression of PTSS, we hypothesize that these variations will be reflected in speech features, including acoustic, temporal, and linguistic aspects, and will significantly differ between individuals with and without PTSD.

## 2 Materials and methods

### 2.1 Participants

The data utilized in the presented analysis originated from a secondary examination of the DAIC dataset, specifically the DAIC_WOZ (Gratch et al., 2014). The original DAIC project was initiated at the University of Southern California and obtained ethical approval from the USC ethical board (UP-11-00342). Data from the DAIC_WOZ were gathered from individuals who underwent assessments for PTSD and MDD, alongside age- and sex-matched control subjects. Participant recruitment occurred through online advertisements posted on Craigslist.org and on-site at a U.S. Veterans Facility in Southern California. The inclusion criteria for patients required participants to be aged 18–65, with prior diagnoses of PTSD or major depressive disorder (MDD), and to be fluent English speakers. All interviews were conducted in English, and participants were interviewed either at the USC Institute for Creative Technologies (ICT) in Los Angeles or at the U.S. veterans’ site. Prior to their involvement in the study, all participants provided informed consent.

### 2.2 Clinical assessment

During the assessment process, participants completed several self-reported questionnaires. To assess PTSD, the PTSD Checklist - Civilian Version [PCL-C (Weathers et al., 1994)] was administered once. The questionnaire consists of 17 items, inquiring about experiencing symptoms within the last month such as “Repeated, disturbing memories, thoughts, or images of a stressful experience from the past.” Answers are given on a Five-point Likert scale with descriptions ranging from “Not at all” (1) to “Extremely” (5). A score between 17 and 29 shows little to no severity. Scores of 28 or higher are indicative of a clinically significant number of symptoms. The reliability of the PCL-C has been confirmed in various study samples with consistent Cronbach’s ɑ above 0.8 (Wilkins et al., 2011).

Additionally, the Patient Health Questionnaire [PHQ-8 (Kroenke et al., 2001)] was used for assessing depression. The self-reported measure consists of eight Likert type items with answers ranging from 0 (“Not at all”) to 3 (“Nearly every day”), referring to the presence of the respective symptoms during the last 2 weeks. The eight items correspond to the first eight symptoms of the DSM-IV diagnostic criteria for MDD (American Psychiatric Association, 1994), the valid classification system at the time of participant recruitment. Examples for the symptoms questioned are “Little interest or pleasure in doing things” or “Feeling down, depressed, or hopeless.” Participants with a score of 10 or greater are considered as having clinically relevant depressive symptoms. Cronbach’s ɑ for the PHQ-8 has been demonstrated to be consistently 0.8 or above in various samples and languages (De La Torre et al., 2023). In this sample, meeting the criteria for PTSD or depression required more than just scoring above the cut-off points as described above. This involved an algorithmic calculation in which specific questionnaire criteria had to be met to qualify for the respective disorder, even if the overall score exceeded the cut-offs. This was undertaken to reflect the fulfillment of core symptoms and additional symptoms of the respective disorders. For the PCL-C, this ensured the core symptoms of intrusive recollections, avoidance/numbing symptoms, and hyper-arousal symptoms to be present, as required according to the DSM-IV diagnostic criteria (American Psychiatric Association, 1994). For the PHQ-8 a cut-off of 10 as well as a minimum of 4 different depressive symptoms had to be fulfilled to ensure the presence of clinically relevant depressive symptoms. A PHQ-8 score of 10 or greater serves as a valid approximation to diagnose MDD by a semi-structured interview such as the SCID (First and Gibbon, 2004) with sensitivity and specificity of >0.8 (Wu et al., 2020). The respective algorithms can be found in the Supplementary material.

### 2.3 Recording setup and transcription

The interviews were conducted using different modalities. Participants underwent interviews employing a Wizard-of-Oz technique, where an animated virtual interviewer named Ellie was utilized. Human interviewers controlled Ellie from a separate room, and this process typically lasted between 5 and 20 min. An alternative method involved automated interviews, where Ellie conducted interviews in a fully autonomous mode, with durations ranging from 15 to 25 min.

Throughout the interviews, participants were posed a series of questions. Some of these questions aimed to evoke emotional involvement, such as: “How are you doing today?”; “When was the last time you argued with someone, and what was it about?”; “How did you feel in that moment?”; “Tell me about an event or something that you wish you could erase from your memory.”; “Tell me about the last time you felt really happy.”; “How would your best friend describe you?”; “Have you noticed any changes in your behavior or thoughts lately?”; and “What’s one of your most memorable experiences?”.

The interviews recorded with the Wizard-of-Oz technique were segmented and transcribed using the EUDICO Linguistic Annotator (ELAN) tool from the Max Planck Institute for Psycholinguistics (Brugman and Russel, 2004). For a detailed description of the process please refer to Gratch et al. (2014).

### 2.4 Data processing and statistical analysis

We conducted a comprehensive analysis of various speech components which were previously associated with symptoms of PTSD (Papini et al., 2015; Marmar et al., 2019). Speech features were further grouped into categories (see Supplementary Table S1 for feature groups and associated features). The acoustic features for these analyses were extracted with the openSMILE software using the Geneva Minimalistic Acoustic Parameter Set (GeMAPS, Eyben et al., 2016) and are listed in Supplementary Table S1 in the categories *Energy, Frequency*, and *Voiced/Unvoiced*.

Additionally, we used features defined by our working group pertaining to *temporal* aspects of speech (König et al., 2019). These refer to timing-related characteristics of spoken language, including the duration, rhythm, and timing patterns (Zellner, 1994).

Furthermore we put a focus on linguistic features such as pronouns, adjectives, adverbs, and conjunctions. These categories are listed in the categories *Lexical Richness* and *Word Types*. The features within these categories were defined by our working group (Lindsay et al., 2021).

Lastly, to capture emotional response, we investigated *Sentiment* (positive or negative valence), which has been linked to PTSD (Jaeger et al., 2014; Sawalha et al., 2022). To assess these linguistic aspects, we used an external Python library called Stanza (Qi et al., 2020), which is based on large language models (LLM), specifically neural networks that utilize contextualized word embeddings. It uses pre-trained language models to determine the types of words used and also for each sentence whether they are in a positive, neutral or negative tone. Stanza is an open-source Python natural language processing (NLP) toolkit supporting 66 human languages developed by the Stanford NLP Group (Socher et al., 2013). It has been demonstrated to be the best among eight other NLP tools to automatically conduct linguistic extractions with an accuracy of up to 0.92 to extract noun phrases (Danenas and Skersys, 2022).

Linguistic and acoustic features were extracted from the participants’ audio-recorded answers provided in response to the questions asked by the virtual avatar. To compute the features, the extraction scripts were implemented in Python 3.9, based on our own speech processing library (“Sigma”) and openSMILE. The extraction code is available upon reasonable request as described below.

### 2.5 Statistical analysis

Outliers were removed if their feature values exceeded five standard deviations from the mean. If an outlier was detected in one of the participant’s features, the entire data for that participant was excluded from further analysis. Since our analysis focused on PTSD, participants who could be categorized as “depression only” were excluded.

Using the implementation from the Python package scipy.stats (v1.11.4, linux v5.10.0), we assessed differences in PCL-C scores between PTSD females and males for each question with Mann–Whitney U tests. Common Language Effect (CLE) *d* served as a measure for effect size. CLE indicates the probability that a randomly selected sample from one group has a larger value than a randomly selected sample from the other group. In that sense, values around 0.5 are generally considered to indicate no difference between groups, values between 0.6 and 0.7, or between 0.3 and 0.4 represent a moderate effect, and values of 0.8 and higher, or 0.2 and lower indicate a large effect size. Since not all participants were asked all questions due to the naturalistic flow of the conversation, subsamples were formed based on the participants who answered each specific question. The PCL-C, as a single self-reported measure assessed once per participant (see Section 2.2, *Clinical Assessment*), was analyzed within these subsamples to evaluate differences between males and females for the corresponding questions. Additionally, we calculated correlation coefficients (Spearman’s *ρ*) between speech features and the PCL-C, stratified by sex. Finally, a linear regression model was used to investigate PTSD severity as assessed by the PCL-C, integrating speech features, sex, and their interaction. Since male and female participants differed significantly in age, and age-related speech changes have been documented (Bóna, 2014; Markova et al., 2016; Rojas et al., 2020; Taylor et al., 2020), all speech feature analyses were adjusted for age. For the Mann–Whitney *U* tests, we computed the group comparisons on the residuals of a linear model predicting the corresponding speech feature from age. For the correlations, we computed partial Spearman-Rank Sum correlations partialling out the effects of age. Lastly, for the linear model assessing the interaction effect between speech features and sex, we included age as a predictor in the model to account for its effects.

All *p*-values reported were adjusted for multiple hypothesis testing using the Benjamini-Hochberg procedure (Benjamini and Hochberg, 1995) clustered in categories as presented in Supplementary Table S1.

## 3 Results

### 3.1 Demographics

The sample comprised a total of *N* = 31 participants (13 female). Due to the variability in the questions asked, different numbers of transcripts were available, as not all participants were posed the same questions. Demographic and clinical data, including age, sex, and PCL-C of PTSD individuals scores are detailed in Table 1.

**Table 1:** Demographic characteristics of PTSD individuals.

Variable	Male PTSD (*n* = 18)	Female PTSD (*n* = 13)	*p*-value (Mann–Whitney)
Age	34.61 (11.43)	45.08 (11.96)	0.026
Years of education	14.5 (1.95)	14.75 (1.6)	0.692
PCL-C score	53.28 (7.55)	62.85 (11.2)	0.018
Word count	29.89 (34.22)	29.62 (29.53)	0.904
Word count min	4	3	
Word count max	146	116	

*R*^2^ = coefficient of determination (goodness of fit).

Values represent means with standard deviations in parentheses. PCL-C = PTSD Checklist – Civilian Version.

### 3.2 Group differences in PCL-C between male and female individuals

Out of the nine questions asked, only for the question “memorable experience” in the PTSD group, significant differences in PCL-C scores between males (*n* = 18) and females (*n* = 13) were observed (Table 2). For this question, females scored higher, indicating greater PTSD severity in this group (CLE *d =* 0.248, *p* = 0.019). Since our research questions were based on differences in expression of PTSS, all subsequent analyses were conducted on the speech features assessed with this question.

**Table 2:** Mann–Whitney *U* test results for differences in PCL-C scores between male and female PTSD individuals.

Variable	*p*-value	Effect size (common language effect *d*)
Travel	0.975	0.505
Dream job	0.391	0.424
Memorable experience	0.019	0.248
Behavior changes	0.621	0.446
BF describe	0.404	0.414
Happy last time	0.485	0.579
How doing	0.567	0.450
Last argument	0.565	0.448
Memory erase	0.556	0.591

Effect sizes are reported as common language effect *d* values.

### 3.3 Differences in speech features between male and female PTSD patients

Examining speech features for the question “memorable experience” in the PTSD individuals, several attributes exhibited significant differences in acoustic features (Table 3). The highest effect size was found for the frequency F2 standard deviation (CLE *d* = 0.816, *p* < 0.05), with males having higher values than females. This feature demonstrates the variability in the vocal tract’s resonance during speech, higher values indicating greater variability.

**Table 3:** Group differences (Mann–Whitney *U* test) between male and female PTSD patients.

Variable	Effect size (common language effect *d*)	*p*	Adjusted *p*
HNRdBACF_sma3nz_amean	0.081	<0.001	0.001
F0semitoneFrom27.5Hz_sma3nz_amean	0.073	<0.001	0.002
F2frequency_sma3nz_stddevNorm	0.816	0.003	0.039

Variables are ordered by descending adjusted *p*-value and effect size. The adjusted *p*-values are computed using the Benjamini–Hochberg procedure. Only features with *p* < 0.05 are reported here; see Supplementary Table S2 for full results.

Another feature significantly differing between males and females was the harmonic to noise ratio (HNR) with males showing a lower value than females (CLE *d* = 0.073, *p* < 0.01). The HNR measures the proportion of harmonic (periodic) components to noise (aperiodic) components in a voice signal.

Furthermore, the fundamental frequency F0 showed lower values (CLE *d* = 0.073, *p* < 0.01) in males than females. This variable represents the average fundamental frequency (pitch) of speech.

### 3.4 Correlations of speech features and PCL-C stratified by sex

Correlations between specific speech features and PCL-C scores revealed the strongest association for the variable *Loudness Standard Deviation* in male participants (*ρ* = 0.66, *p* < 0.01). Other variables in the male subsample showed correlation coefficients of less than 0.5. In female participants, variables related to sentiment, acoustics, and grammatical structures exhibited the highest correlation values, ranging from −0.53 to −0.66. However, none of these variables, in either males or females, remained statistically significant after adjusting for multiple hypothesis testing (*p* > 0.2, respectively). A comprehensive list of results is provided in Supplementary Tables S3, S4.

### 3.5 Linear regression model

**Figure 1:**
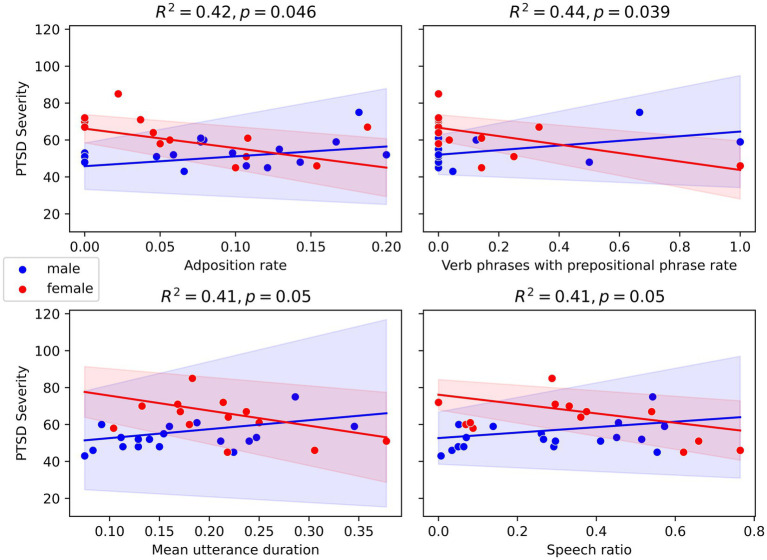
Scatter plot of linear regression models with standard error of selected speech features.

For the linear model (age, speech feature, sex, and the interaction of speech features and sex, dependent variable: PCL-C score), within the group of PTSD participants, we found significant interaction effects for several features, such as the variable “verb_phrase_with_vbg_pp_rate.” This feature describes verb phrases headed by a gerund or present participle and followed by a prepositional phrase, e.g., “She was eating ice cream by the river” (*p <* 0.05). In our data, males tend to use more of these phrases as their symptomatology increases, whereas the inverse is true for females. These inverse associations (positive for males, negative for females) were observed for all further significant features (*p* < 0.05, respectively). These variables included the adposition rate (frequency of prepositions and postpositions used in speech), the mean utterance duration and speech ratio (proportion of speech produced by a participant relative to the total speech in the conversation). Figure 1 illustrates the interaction effects of the linear regression models. For the remainder of the features, no significant interactions were observed. Supplementary Table S5 depicts all feature values.

## 4 Discussion

In this study, we investigated differences in speech features among male and female PTSD patients, focusing on responses to the prompt “What’s one of your most memorable experiences?” Significant distinctions emerged primarily in acoustic attributes, notably in the F2 formant frequency and the HNR. Linear regression models revealed significant interaction effects for speech features such as verb phrase usage, adposition rate, mean utterance duration, and speech ratio, with males showing positive associations and females showing inverse associations.

The reported higher standard deviations of the F2 frequency in males compared to females suggest that male PTSD patients exhibit more variability in their vowel production. This increased variability could be due to factors such as increased psychological stress, physiological differences, or differential impacts of trauma on the vocal mechanism.

In our data, we found a lower HNR in male PTSD individuals compared to females. A higher HNR indicates a cleaner, more periodic voice signal, often associated with healthier vocal function. Conversely, a lower HNR suggests a voice with more noise, potentially indicating vocal strain or pathology. A lower HNR in males can thus be interpreted as an indication of higher levels of vocal noise. This suggests that male PTSD patients might experience more vocal strain or issues compared to their female counterparts. Factors such as increased psychological stress, physiological differences, and sociocultural aspects might contribute to this disparity. Studies have shown that stress and psychological disorders can significantly affect vocal function (Dietrich and Verdolini Abbott, 2012; Holmqvist et al., 2013), reflecting the overall impact on vocal health. Our finding of a lower fundamental frequency (F0) in males, perceived as pitch, is not surprising given the natural differences in vocal anatomy and physiology between sexes, with males on average having larger vocal cords and a deeper voice compared to females.

The correlation analyses conducted in our study suggest that various speech features exhibit notable correlations with PCL-C scores, with positive associations in males and a mix of negative and positive associations in females. The strongest correlation was found for the variable *Loudness Standard Deviation* in males (*ρ* = 0.66, *p* < 0.01), indicating that male individuals with more fluctuating speech volume experience more symptomatology, an effect that was not observed in the female subsample. One possible explanation for this sex difference is that males with PTSD may experience heightened emotional reactivity and difficulty regulating their emotional expressions, which could lead to more pronounced fluctuations in speech volume. Indeed it has been demonstrated that male PTSD patients report significantly higher rates of reckless or self-destructive behavior compared to females (Murphy et al., 2019), suggesting higher levels of outwardly directed emotional instability, which might in turn be reflected in their speech. None of the other speech features in either males or females showed statistically significant correlations after adjusting for multiple hypothesis testing (*p* > 0.2). To our knowledge, no published data describe sex-specific differences in speech loudness among individuals with PTSD.

Furthermore, we investigated the interaction effects of speech features and sex on PCL-C scores among PTSD participants using a linear regression model. Our findings revealed significant interaction effects for a variable describing verb phrases headed by a gerund or present participle and followed by a prepositional phrase (*p* < 0.05). Another significant finding was the adposition rate (*p* < 0.05), indicating that these speech features differ by sex among PTSD patients. These results suggest that the use of more complex or specific grammatical structures, such as these verb phrases and adpositions, may be influenced by sex in PTSD patients. Males, for example, may show greater specificity in their speech, while females may tend toward vaguer expressions. Brown et al. (2014) found that PTSD patients often use less specific language when recalling autobiographical memories. It is possible that sex differentially affects this tendency, with males and females exhibiting different patterns of speech specificity as a result of distinct cognitive and emotional responses to PTSD (Ramikie and Ressler, 2018).

Additionally, we observed significant interaction effects for utterance duration and speech ratio (*p* = 0.05, respectively), suggesting potential sex-specific variations in these features. Specifically, males with higher PCL-C scores tended to have a higher speech ratio and longer mean utterances, whereas females with higher PCL-C scores showed a lower speech ratio and shorter utterances. These differences may reflect distinct communicative strategies or emotional processing in response to PTSD symptoms. Previous studies have noted that PTSD can impact speech differently between men and women. For instance, Crevier et al. (2014) highlighted that women with PTSD exhibit different interpersonal communication patterns compared to men (Crevier et al., 2014). These findings did not specifically refer to grammatical structure but rather to speech content, however. However, these findings support the notion that not just voice but specifically language in PTSD patients might differ between sexes generally.

There are several limitations to this study. The small sample size may limit generalizability, increasing the likelihood of sampling bias and reducing statistical power. Future studies should include larger, more diverse samples and ensure consistent characterization of PTSD severity across participants. Additionally, we cannot fully exclude the possibility that the effects we observed reflect natural sex differences in voice and speech, rather than differences specific to PTSD expression between males and females. Also, potential confounders, such as smoking, which can affect voice parameters through changes to the vocal cords and respiratory system (Wei et al., 2024) were not controlled for in this study. Lastly, PTSD diagnoses were based on self-report questionnaires rather than clinical interviews, which may have introduced biases and reduced diagnostic precision.

Our findings unveil sex-related variations in the expression of PTSD severity through speech, suggesting contrasting effects based on sex in acoustic and grammatical features. These novel insights underscore the importance of considering sex-specific expressions of behavioral symptoms in developing digital speech biomarkers for diagnostic and monitoring purposes in PTSD and psychiatry at large. To translate these findings into clinical practice responsibly, it is essential to develop tailored algorithms that account for these sex-based differences, ensuring that speech-based assessments are equally sensitive and accurate for both men and women. Future research should prioritize the inclusion of larger and more representative samples, encompassing diverse demographic and clinical profiles, to ensure findings are broadly applicable. We recommend that our findings be reproduced and validated in external cohorts. Additionally, standardized approaches to defining and assessing PTSD severity are crucial to facilitate comparisons across studies and optimize model performance. By integrating these considerations, translational efforts can move toward creating robust, equitable, and clinically effective speech-based tools for mental health care.

## Data Availability

Publicly available datasets were analyzed in this study. This data can be found: https://dcapswoz.ict.usc.edu/.
