# Supplementary material for: Sex differences in PTSD speech biomarkers assessed by virtual agent-induced conversations
Source: Front Psychol. 2025 Apr 28;16:1509206. doi: 10.3389/fpsyg.2025.1509206 (PMC12066298; doi:10.3389/fpsyg.2025.1509206)
Supplement: Supplementary file 1 [file Table_1.docx]

*Table S1*

Feature categorization and explanation

| Feature group | Feature | Explanation |
| --- | --- | --- |
| Energy |  |  |
|  | HNRdBACF_sma3nz_amean | Mean Harmonic-to-Noise Ratio (HNR) in decibels, calculated using the cepstral analysis of the acoustic signal. |
|  | HNRdBACF_sma3nz_stddevNorm | Standard deviation of HNRdBACF_sma3nz_amean. |
|  | loudness_sma3_amean | Mean speech loudness. |
|  | loudness_sma3_pctlrange0-2 | Range of 20th to 80th percentile of loudness_sma3_amean. |
|  | loudness_sma3_percentile20.0 | 20th percentile of loudness_sma3_amean. |
|  | loudness_sma3_percentile50.0 | 50th percentile of loudness_sma3_amean. |
|  | loudness_sma3_percentile80.0 | 80th percentile of loudness_sma3_amean |
|  | loudness_sma3_stddevNorm | Standard deviation of loudness_sma3_amean. |
| Frequency |  |  |
|  | F0semitoneFrom27.5Hz_sma3nz_amean | F0 on a semitone frequency scale (perceived as pitch), starting at 27.5 Hz. |
|  | F0semitoneFrom27.5Hz_sma3nz_meanFallingSlope | Average rate at which the F0 semitone from 27.5 Hz decreases over a certain period of time. |
|  | F0semitoneFrom27.5Hz_sma3nz_meanRisingSlope | Average rate at which the F0 semitone from 27.5 Hz increases over a certain period of time. |
|  | F0semitoneFrom27.5Hz_sma3nz_pctlrange0-2 | Range of 20th to 80th percentile of F0semitoneFrom27.5Hz_sma3nz_amean. |
|  | F0semitoneFrom27.5Hz_sma3nz_percentile20.0 | 20th percentile of F0semitoneFrom27.5Hz_sma3nz_amean. |
|  | F0semitoneFrom27.5Hz_sma3nz_percentile50.0 | 50th percentile of F0semitoneFrom27.5Hz_sma3nz_amean. |
|  | F0semitoneFrom27.5Hz_sma3nz_percentile80.0 | 80th percentile of F0semitoneFrom27.5Hz_sma3nz_amean. |
|  | F0semitoneFrom27.5Hz_sma3nz_stddevFallingSlope | Standard deviation of F0semitoneFrom27.5Hz_sma3nz_stddevFallingSlope. |
|  | F0semitoneFrom27.5Hz_sma3nz_stddevNorm | Standard deviation of F0semitoneFrom27.5Hz_sma3nz_amean. |
|  | F0semitoneFrom27.5Hz_sma3nz_stddevRisingSlope | Standard deviation of F0semitoneFrom27.5Hz_sma3nz_meanRisingSlope. |
|  | F1bandwidth_sma3nz_amean | Mean bandwidth of F1 formant. |
|  | F1bandwidth_sma3nz_stddevNorm | Standard deviation of F1bandwidth_sma3nz_stddevNorm. |
|  | F1frequency_sma3nz_amean | Mean frequency of F1 formant. |
|  | F1frequency_sma3nz_stddevNorm | Standard deviation of F1frequency_sma3nz_amean. |
|  | F2bandwidth_sma3nz_amean | Mean bandwidth of F2 formant. |
|  | F2bandwidth_sma3nz_stddevNorm | Standard deviation of F2bandwidth_sma3nz_amean. |
|  | F2frequency_sma3nz_amean | Mean frequency of F2 formant. |
|  | F2frequency_sma3nz_stddevNorm | Standard deviation of F2frequency_sma3nz_amean. |
|  | F3bandwidth_sma3nz_amean | Mean bandwidth of F3 formant. |
|  | F3bandwidth_sma3nz_stddevNorm | Standard deviation of F3bandwidth_sma3nz_amean. |
|  | F3frequency_sma3nz_amean | Mean frequency of F3 formant. |
|  | F3frequency_sma3nz_stddevNorm | Standard deviation of F3frequency_sma3nz_amean. |
|  | jitterLocal_sma3nz_amean | Deviations in individual consecutive F0 period lengths (perceived as uneven or irregular voice). |
|  | jitterLocal_sma3nz_stddevNorm | Standard deviation of jitterLocal_sma3nz_amean. |
| Lexical Richness |  |  |
|  | brunets_index | Brunet’s Index, measure of lexical diversity. |
|  | honore_stat | Honoré’s statistic, measure to assess the lexical richness or diversity. |
|  | mean_frazier_depth | Mean Frazier depth (measure of complexity of syntactic structures) over all sentences. |
|  | mean_yngve_depth | Yngve depth, level of nested or embedded clauses within a sentence. |
|  | word_frequency_max | The maximum word frequency of all tokens. |
|  | word_frequency_mean | Mean of word frequency of all tokens. |
|  | word_frequency_min | The minimum word frequency of all tokens. |
| Sentiment |  |  |
|  | mean_sentiment | Average emotional valence of the sentences (indicating if the whole answer was in general more positive, neutral, or negative). |
|  | negative_sentence_ratio | Number of sentences that are labeled as negative in relation to all sentences. |
|  | neutral_sentence_ratio | Number of sentences that are labeled as neutral in relation to all sentences. |
|  | positive_sentence_ratio | Number of sentences that are labeled as positive in relation to all sentences. |
| Temporal |  |  |
|  | duration | Length of audio recording. |
|  | number_of_pauses | Number of pauses in between speech segments based on speech intervals. |
|  | pause_durations_mean | Mean length of pauses. |
|  | pause_durations_std | Standard deviation of pause duration. |
|  | pause_durations_sum | Sum of pause lengths over whole utterance. |
|  | pause_rate | Frequency of pauses within a spoken utterance. |
|  | speech_ratio | Ratio of utterance to duration. |
|  | utterance_durations_mean | Mean length of utterances. |
|  | utterance_durations_std | Standard deviation of utterance durations. |
|  | utterance_durations_sum | Sum of utterance durations. |
| Voiced/Unvoiced |  |  |
|  | MeanUnvoicedSegmentLength | Mean length of unvoiced segments. |
|  | MeanVoicedSegmentLengthSec | Mean length of voiced segments. |
|  | StddevVoicedSegmentLengthSec | Standard deviation of the mean length of voiced segments. |
|  | StddevUnvoicedSegmentLength | Standard deviation of the mean length of unvoiced segments. |
|  | VoicedSegmentsPerSec | Number of voiced segments per second. |
| Word Types |  |  |
|  | adjective_rate | Relative frequency (ratio to total words spoken) of adjectives used. |
|  | adposition_rate | Ratio of adpositions used. |
|  | adverb_rate | Ratio of adverbs used. |
|  | conjunction_rate | Ratio of conjunctions used. |
|  | determiner_rate | Ratio of determiners (articles, demonstratives, possessives) used. |
|  | inflected_verb_rate | Ratio of inflected verbs used. |
|  | noun_rate | Ratio of nouns used. |
|  | number_deictic_terms | Frequency of deictic terms used. |
|  | pronoun_rate | Ratio of pronouns used. |
|  | proper_noun_rate | Ratio of proper nouns used. |
|  | verb_phrase_with_aux_and_vp_rate | Ratio of verb phrases that include both auxiliary verbs and main verbs (e.g. She has finished her homework). |
|  | verb_phrase_with_aux_rate | Ratio of verb phrases that include an auxiliary verb. |
|  | verb_phrase_with_vbg_rate | Ratio of verb phrases that include a gerund or present participle. |
|  | verb_phrase_with_vbg_pp_rate | Number of times (ratio) a Verb Phrase (VP) is headed by a VBG (gerund or present participle, e.g. eating ice cream) and takes a prepositional phrase (PP) (e.g. by the river) relative to the total number of verb phrases in the whole transcript using the constituency parse tree CFG rules. |

*Table S2*

Group differences (Mann-Whitney U test) between female and male PTSD patients.

|  | p | Effect size | Adj. p | M vs F |
| --- | --- | --- | --- | --- |
| HNRdBACF_sma3nz_amean | <0.001 | 0.081 | 0.001 | < |
| F0semitoneFrom27.5Hz_sma3nz_amean | <0.001 | 0.073 | 0.002 | < |
| F2frequency_sma3nz_stddevNorm | 0.003 | 0.816 | 0.039 | > |
| F1frequency_sma3nz_stddevNorm | 0.008 | 0.786 | 0.062 | > |
| F2bandwidth_sma3nz_stddevNorm | 0.019 | 0.248 | 0.073 | < |
| F3bandwidth_sma3nz_stddevNorm | 0.019 | 0.248 | 0.073 | < |
| logRelF0-H1-H2_sma3nz_stddevNorm | 0.021 | 0.252 | 0.073 | > |
| F3frequency_sma3nz_stddevNorm | 0.019 | 0.752 | 0.073 | > |
| mean_sentiment | 0.026 | 0.261 | 0.105 | < |
| positive_sentence_ratio | 0.144 | 0.342 | 0.144 | < |
| negative_sentence_ratio | 0.118 | 0.669 | 0.144 | < |
| neutral_sentence_ratio | 0.114 | 0.671 | 0.144 | > |
| logRelF0-H1-H2_sma3nz_amean | 0.069 | 0.303 | 0.18 | < |
| F2frequency_sma3nz_amean | 0.075 | 0.308 | 0.18 | < |
| F3bandwidth_sma3nz_amean | 0.069 | 0.697 | 0.18 | > |
| F3amplitudeLogRelF0_sma3nz_amean | 0.222 | 0.368 | 0.434 | < |
| logRelF0-H1-A3_sma3nz_stddevNorm | 0.289 | 0.385 | 0.434 | < |
| F2amplitudeLogRelF0_sma3nz_amean | 0.289 | 0.385 | 0.434 | < |
| F1amplitudeLogRelF0_sma3nz_amean | 0.307 | 0.389 | 0.434 | < |
| F0semitoneFrom27.5Hz_sma3nz_stddevNorm | 0.307 | 0.389 | 0.434 | < |
| logRelF0-H1-A3_sma3nz_amean | 0.289 | 0.615 | 0.434 | > |
| F3amplitudeLogRelF0_sma3nz_stddevNorm | 0.238 | 0.628 | 0.434 | > |
| utterance_durations_sum | 0.089 | 0.316 | 0.444 | < |
| utterance_durations_mean | 0.089 | 0.316 | 0.444 | < |
| F1amplitudeLogRelF0_sma3nz_stddevNorm | 0.347 | 0.603 | 0.462 | > |
| utterance_durations_std | 0.193 | 0.359 | 0.475 | < |
| pause_durations_sum | 0.207 | 0.363 | 0.475 | < |
| number_of_pauses | 0.238 | 0.372 | 0.475 | < |
| F3frequency_sma3nz_amean | 0.389 | 0.406 | 0.492 | < |
| loudness_sma3_percentile20.0 | 0.123 | 0.333 | 0.493 | < |
| F1frequency_sma3nz_amean | 0.435 | 0.415 | 0.497 | < |
| F2bandwidth_sma3nz_amean | 0.435 | 0.585 | 0.497 | > |
| F2amplitudeLogRelF0_sma3nz_stddevNorm | 0.459 | 0.581 | 0.501 | > |
| duration | 0.368 | 0.402 | 0.51 | < |
| speech_ratio | 0.389 | 0.406 | 0.51 | < |
| pause_durations_mean | 0.435 | 0.415 | 0.51 | < |
| pause_rate | 0.459 | 0.419 | 0.51 | < |
| verb_phrase_with_vbg_pp_rate | 0.089 | 0.316 | 0.606 | < |
| adverb_rate | 0.128 | 0.335 | 0.606 | < |
| proper_noun_rate | 0.186 | 0.643 | 0.606 | > |
| determiner_rate | 0.18 | 0.645 | 0.606 | > |
| loudness_sma3_amean | 0.271 | 0.38 | 0.642 | < |
| loudness_sma3_percentile80.0 | 0.412 | 0.41 | 0.642 | < |
| loudness_sma3_percentile50.0 | 0.562 | 0.436 | 0.642 | < |
| loudness_sma3_pctlrange0-2 | 0.562 | 0.436 | 0.642 | < |
| HNRdBACF_sma3nz_stddevNorm | 0.562 | 0.564 | 0.642 | > |
| word_frequency_min | 0.186 | 0.357 | 0.645 | < |
| word_frequency_mean | 0.193 | 0.359 | 0.645 | < |
| word_frequency_max | 0.459 | 0.419 | 0.645 | > |
| brunets_index | 0.562 | 0.436 | 0.645 | < |
| mean_frazier_depth | 0.645 | 0.551 | 0.645 | > |
| honore_stat | 0.509 | 0.573 | 0.645 | > |
| mean_yngve_depth | 0.368 | 0.598 | 0.645 | > |
| F1bandwidth_sma3nz_stddevNorm | 0.734 | 0.462 | 0.766 | < |
| VoicedSegmentsPerSec | 0.167 | 0.35 | 0.826 | < |
| StddevVoicedSegmentLengthSec | 0.617 | 0.444 | 0.826 | < |
| StddevUnvoicedSegmentLength | 0.826 | 0.526 | 0.826 | > |
| MeanVoicedSegmentLengthSec | 0.734 | 0.538 | 0.826 | > |
| MeanUnvoicedSegmentLength | 0.484 | 0.577 | 0.826 | > |
| number_deictic_terms | 0.447 | 0.417 | 0.867 | > |
| adjective_rate | 0.447 | 0.417 | 0.867 | < |
| inflected_verb_rate | 0.562 | 0.436 | 0.867 | < |
| verb_phrase_with_vbg_rate | 0.631 | 0.447 | 0.867 | < |
| noun_rate | 0.704 | 0.457 | 0.867 | < |
| conjunction_rate | 0.734 | 0.462 | 0.867 | > |
| verb_phrase_with_aux_rate | 0.603 | 0.558 | 0.867 | < |
| loudness_sma3_stddevNorm | 0.889 | 0.483 | 0.889 | > |
| pronoun_rate | 0.826 | 0.474 | 0.895 | < |
| pause_durations_std | 0.92 | 0.487 | 0.92 | < |
| F1bandwidth_sma3nz_amean | 0.92 | 0.513 | 0.92 | < |
| adposition_rate | 0.984 | 0.496 | 0.984 | > |

Note. Variables are listed in descending order for adjusted *p* value and effect size. *p*=unadjusted *p* value; Adj. *p*=*p* value adjusted for multiple hypothesis testing according to Benjamini-Hochberg procedure; M=male; F=female.

*Table S3*

Correlation coefficients of speech features and PCL-scores among male PTSD participants.

|  | Spearman’s ρ | p | Adj. p |
| --- | --- | --- | --- |
| loudness_sma3_stddevNorm | 0.657 | 0.004 | 0.033 |
| speech_ratio | 0.399 | 0.113 | 0.282 |
| utterance_durations_mean | 0.415 | 0.097 | 0.282 |
| utterance_durations_std | 0.435 | 0.081 | 0.282 |
| utterance_durations_sum | 0.488 | 0.047 | 0.282 |
| number_of_pauses | 0.342 | 0.18 | 0.299 |
| pause_durations_std | 0.343 | 0.178 | 0.299 |
| pause_durations_mean | -0.237 | 0.36 | 0.459 |
| pause_rate | 0.234 | 0.367 | 0.459 |
| mean_sentiment | -0.312 | 0.222 | 0.461 |
| positive_sentence_ratio | -0.244 | 0.346 | 0.461 |
| neutral_sentence_ratio | 0.248 | 0.338 | 0.461 |
| pause_durations_sum | 0.207 | 0.425 | 0.472 |
| MeanUnvoicedSegmentLength | -0.215 | 0.407 | 0.509 |
| MeanVoicedSegmentLengthSec | 0.221 | 0.394 | 0.509 |
| VoicedSegmentsPerSec | 0.224 | 0.388 | 0.509 |
| StddevVoicedSegmentLengthSec | 0.306 | 0.232 | 0.509 |
| loudness_sma3_amean | 0.245 | 0.343 | 0.548 |
| loudness_sma3_percentile50.0 | 0.253 | 0.328 | 0.548 |
| loudness_sma3_percentile80.0 | 0.296 | 0.248 | 0.548 |
| loudness_sma3_pctlrange0-2 | 0.35 | 0.169 | 0.548 |
| StddevUnvoicedSegmentLength | -0.148 | 0.571 | 0.571 |
| adverb_rate | -0.451 | 0.069 | 0.713 |
| proper_noun_rate | -0.225 | 0.384 | 0.713 |
| adjective_rate | -0.143 | 0.584 | 0.713 |
| verb_phrase_with_aux_rate | -0.136 | 0.604 | 0.713 |
| pronoun_rate | -0.103 | 0.695 | 0.713 |
| inflected_verb_rate | 0.097 | 0.713 | 0.713 |
| verb_phrase_with_vbg_rate | 0.103 | 0.693 | 0.713 |
| conjunction_rate | 0.146 | 0.577 | 0.713 |
| number_deictic_terms | 0.152 | 0.56 | 0.713 |
| noun_rate | 0.185 | 0.476 | 0.713 |
| verb_phrase_with_vbg_pp_rate | 0.187 | 0.472 | 0.713 |
| adposition_rate | 0.205 | 0.431 | 0.713 |
| determiner_rate | 0.391 | 0.121 | 0.713 |
| F3amplitudeLogRelF0_sma3nz_stddevNorm | -0.386 | 0.126 | 0.754 |
| F2amplitudeLogRelF0_sma3nz_stddevNorm | -0.379 | 0.134 | 0.754 |
| F1amplitudeLogRelF0_sma3nz_stddevNorm | -0.316 | 0.216 | 0.754 |
| F3frequency_sma3nz_stddevNorm | 0.295 | 0.25 | 0.754 |
| F1amplitudeLogRelF0_sma3nz_amean | 0.295 | 0.251 | 0.754 |
| F2amplitudeLogRelF0_sma3nz_amean | 0.303 | 0.237 | 0.754 |
| logRelF0-H1-H2_sma3nz_stddevNorm | 0.308 | 0.23 | 0.754 |
| F3amplitudeLogRelF0_sma3nz_amean | 0.327 | 0.201 | 0.754 |
| F0semitoneFrom27.5Hz_sma3nz_amean | 0.269 | 0.296 | 0.788 |
| duration | 0.053 | 0.839 | 0.839 |
| HNRdBACF_sma3nz_amean | 0.126 | 0.629 | 0.839 |
| F3frequency_sma3nz_amean | -0.193 | 0.458 | 0.865 |
| F1bandwidth_sma3nz_amean | -0.147 | 0.575 | 0.865 |
| F2bandwidth_sma3nz_amean | -0.096 | 0.715 | 0.865 |
| logRelF0-H1-H2_sma3nz_amean | -0.086 | 0.742 | 0.865 |
| logRelF0-H1-A3_sma3nz_amean | -0.079 | 0.762 | 0.865 |
| F2bandwidth_sma3nz_stddevNorm | -0.071 | 0.786 | 0.865 |
| logRelF0-H1-A3_sma3nz_stddevNorm | 0.057 | 0.829 | 0.865 |
| F3bandwidth_sma3nz_amean | 0.063 | 0.811 | 0.865 |
| F1frequency_sma3nz_stddevNorm | 0.064 | 0.807 | 0.865 |
| F1bandwidth_sma3nz_stddevNorm | 0.065 | 0.805 | 0.865 |
| F2frequency_sma3nz_amean | 0.099 | 0.705 | 0.865 |
| F1frequency_sma3nz_amean | 0.155 | 0.553 | 0.865 |
| F3bandwidth_sma3nz_stddevNorm | 0.158 | 0.544 | 0.865 |
| F2frequency_sma3nz_stddevNorm | 0.23 | 0.375 | 0.865 |
| negative_sentence_ratio | 0.039 | 0.882 | 0.882 |
| word_frequency_min | -0.233 | 0.368 | 0.885 |
| word_frequency_mean | -0.147 | 0.575 | 0.885 |
| mean_frazier_depth | -0.05 | 0.848 | 0.885 |
| brunets_index | -0.042 | 0.872 | 0.885 |
| mean_yngve_depth | -0.038 | 0.885 | 0.885 |
| honore_stat | 0.1 | 0.703 | 0.885 |
| word_frequency_max | 0.125 | 0.632 | 0.885 |
| F0semitoneFrom27.5Hz_sma3nz_stddevNorm | -0.032 | 0.902 | 0.902 |
| loudness_sma3_percentile20.0 | 0.004 | 0.987 | 0.987 |
| HNRdBACF_sma3nz_stddevNorm | 0.04 | 0.878 | 0.987 |

Note. Variables are listed in descending order for adjusted *p* value and correlation coefficient. *p*=unadjusted *p* value; Adj. *p*=*p* value adjusted for multiple hypothesis testing according to Benjamini-Hochberg procedure.

*Table S4*

Correlation coefficients of speech features and PCL-scores among female PTSD participants.

|  | Spearman’s ρ | p | Adj. p |
| --- | --- | --- | --- |
| adposition_rate | -0.659 | 0.02 | 0.193 |
| verb_phrase_with_vbg_pp_rate | -0.625 | 0.03 | 0.193 |
| HNRdBACF_sma3nz_stddevNorm | 0.622 | 0.031 | 0.248 |
| mean_sentiment | -0.577 | 0.049 | 0.126 |
| pause_durations_std | 0.559 | 0.059 | 0.435 |
| positive_sentence_ratio | -0.552 | 0.063 | 0.126 |
| loudness_sma3_percentile20.0 | -0.527 | 0.079 | 0.314 |
| pause_durations_mean | 0.474 | 0.119 | 0.435 |
| speech_ratio | -0.462 | 0.131 | 0.435 |
| pause_rate | 0.397 | 0.201 | 0.503 |
| loudness_sma3_stddevNorm | 0.379 | 0.224 | 0.37 |
| loudness_sma3_percentile50.0 | -0.368 | 0.239 | 0.37 |
| noun_rate | -0.356 | 0.256 | 0.996 |
| loudness_sma3_amean | -0.341 | 0.278 | 0.37 |
| loudness_sma3_percentile80.0 | -0.341 | 0.278 | 0.37 |
| negative_sentence_ratio | 0.33 | 0.296 | 0.366 |
| F2frequency_sma3nz_stddevNorm | -0.327 | 0.3 | 0.995 |
| utterance_durations_mean | -0.322 | 0.308 | 0.615 |
| logRelF0-H1-H2_sma3nz_stddevNorm | 0.314 | 0.321 | 0.995 |
| F2bandwidth_sma3nz_stddevNorm | 0.291 | 0.359 | 0.995 |
| neutral_sentence_ratio | 0.287 | 0.366 | 0.366 |
| F0semitoneFrom27.5Hz_sma3nz_stddevNorm | 0.256 | 0.422 | 0.995 |
| adjective_rate | -0.254 | 0.425 | 0.996 |
| conjunction_rate | -0.237 | 0.458 | 0.996 |
| pause_durations_sum | 0.231 | 0.47 | 0.784 |
| F3frequency_sma3nz_stddevNorm | -0.211 | 0.51 | 0.995 |
| word_frequency_mean | -0.19 | 0.554 | 0.813 |
| HNRdBACF_sma3nz_amean | -0.186 | 0.563 | 0.643 |
| inflected_verb_rate | 0.172 | 0.593 | 0.996 |
| number_deictic_terms | 0.162 | 0.615 | 0.996 |
| verb_phrase_with_aux_rate | -0.162 | 0.616 | 0.996 |
| F3bandwidth_sma3nz_amean | -0.161 | 0.618 | 0.995 |
| F2frequency_sma3nz_amean | 0.154 | 0.633 | 0.995 |
| F2bandwidth_sma3nz_amean | 0.153 | 0.636 | 0.995 |
| utterance_durations_sum | -0.15 | 0.643 | 0.819 |
| logRelF0-H1-A3_sma3nz_stddevNorm | 0.147 | 0.649 | 0.995 |
| F2amplitudeLogRelF0_sma3nz_stddevNorm | 0.146 | 0.65 | 0.995 |
| utterance_durations_std | -0.144 | 0.655 | 0.819 |
| mean_yngve_depth | -0.141 | 0.661 | 0.813 |
| F3amplitudeLogRelF0_sma3nz_amean | -0.13 | 0.688 | 0.995 |
| F1amplitudeLogRelF0_sma3nz_stddevNorm | 0.129 | 0.691 | 0.995 |
| F1bandwidth_sma3nz_amean | -0.128 | 0.693 | 0.995 |
| F1amplitudeLogRelF0_sma3nz_amean | -0.127 | 0.693 | 0.995 |
| StddevUnvoicedSegmentLength | -0.125 | 0.7 | 0.988 |
| honore_stat | -0.124 | 0.701 | 0.813 |
| brunets_index | 0.119 | 0.711 | 0.813 |
| loudness_sma3_pctlrange0-2 | -0.119 | 0.713 | 0.713 |
| word_frequency_min | 0.113 | 0.727 | 0.813 |
| F3bandwidth_sma3nz_stddevNorm | 0.112 | 0.73 | 0.995 |
| MeanVoicedSegmentLengthSec | -0.103 | 0.749 | 0.988 |
| F1bandwidth_sma3nz_stddevNorm | 0.102 | 0.752 | 0.995 |
| mean_frazier_depth | 0.095 | 0.769 | 0.813 |
| F2amplitudeLogRelF0_sma3nz_amean | -0.095 | 0.77 | 0.995 |
| F3amplitudeLogRelF0_sma3nz_stddevNorm | 0.089 | 0.784 | 0.995 |
| logRelF0-H1-A3_sma3nz_amean | 0.077 | 0.812 | 0.995 |
| word_frequency_max | 0.076 | 0.813 | 0.813 |
| verb_phrase_with_vbg_rate | -0.073 | 0.821 | 0.996 |
| adverb_rate | 0.065 | 0.84 | 0.996 |
| StddevVoicedSegmentLengthSec | -0.055 | 0.866 | 0.988 |
| logRelF0-H1-H2_sma3nz_amean | -0.051 | 0.876 | 0.995 |
| F0semitoneFrom27.5Hz_sma3nz_amean | 0.048 | 0.883 | 0.995 |
| duration | 0.047 | 0.884 | 0.982 |
| VoicedSegmentsPerSec | 0.043 | 0.895 | 0.988 |
| F3frequency_sma3nz_amean | 0.014 | 0.964 | 0.995 |
| determiner_rate | 0.01 | 0.976 | 0.996 |
| MeanUnvoicedSegmentLength | -0.005 | 0.988 | 0.988 |
| F1frequency_sma3nz_amean | 0.004 | 0.991 | 0.995 |
| number_of_pauses | -0.003 | 0.994 | 0.994 |
| pronoun_rate | -0.002 | 0.994 | 0.996 |
| F1frequency_sma3nz_stddevNorm | -0.002 | 0.995 | 0.995 |
| proper_noun_rate | 0.001 | 0.996 | 0.996 |

Note. Variables are listed in descending order for adjusted *p* value and correlation coefficient. *p*=unadjusted *p* value; Adj. *p*=*p* value adjusted for multiple hypothesis testing according to Benjamini-Hochberg procedure.

*Table S5*

|  | R2 | p | Adj. p |
| --- | --- | --- | --- |
| verb_phrase_with_vbg_pp_rate | 0.223 | 0.003 | 0.039 |
| utterance_durations_mean | 0.194 | 0.007 | 0.05 |
| adposition_rate | 0.193 | 0.007 | 0.046 |
| speech_ratio | 0.177 | 0.01 | 0.05 |
| loudness_sma3_percentile50_0 | 0.156 | 0.014 | 0.112 |
| loudness_sma3_amean | 0.122 | 0.035 | 0.131 |
| loudness_sma3_percentile80_0 | 0.105 | 0.053 | 0.131 |
| HNRdBACF_sma3nz_stddevNorm | 0.085 | 0.087 | 0.131 |
| loudness_sma3_percentile20_0 | 0.067 | 0.089 | 0.131 |
| F3amplitudeLogRelF0_sma3nz_stddevNorm | 0.079 | 0.093 | 0.594 |
| loudness_sma3_pctlrange0_2 | 0.08 | 0.098 | 0.131 |
| F2amplitudeLogRelF0_sma3nz_stddevNorm | 0.076 | 0.1 | 0.594 |
| F1amplitudeLogRelF0_sma3nz_stddevNorm | 0.073 | 0.11 | 0.594 |
| utterance_durations_std | 0.073 | 0.113 | 0.377 |
| F3amplitudeLogRelF0_sma3nz_amean | 0.058 | 0.154 | 0.594 |
| F2amplitudeLogRelF0_sma3nz_amean | 0.056 | 0.163 | 0.594 |
| F3bandwidth_sma3nz_amean | 0.054 | 0.178 | 0.594 |
| MeanUnvoicedSegmentLength | 0.052 | 0.179 | 0.478 |
| F1amplitudeLogRelF0_sma3nz_amean | 0.05 | 0.187 | 0.594 |
| MeanVoicedSegmentLengthSec | 0.05 | 0.191 | 0.478 |
| F0semitoneFrom27_5Hz_sma3nz_stddevNorm | 0.049 | 0.198 | 0.594 |
| mean_yngve_depth | 0.046 | 0.201 | 0.965 |
| logRelF0_H1_A3_sma3nz_amean | 0.044 | 0.224 | 0.597 |
| conjunction_rate | 0.041 | 0.242 | 0.823 |
| negative_sentence_ratio | 0.037 | 0.262 | 0.511 |
| F3frequency_sma3nz_stddevNorm | 0.037 | 0.262 | 0.629 |
| mean_sentiment | 0.029 | 0.291 | 0.511 |
| HNRdBACF_sma3nz_amean | 0.033 | 0.293 | 0.335 |
| utterance_durations_sum | 0.028 | 0.333 | 0.708 |
| F2bandwidth_sma3nz_amean | 0.025 | 0.36 | 0.785 |
| pause_rate | 0.022 | 0.382 | 0.708 |
| positive_sentence_ratio | 0.019 | 0.383 | 0.511 |
| pause_durations_mean | 0.019 | 0.425 | 0.708 |
| proper_noun_rate | 0.017 | 0.448 | 0.823 |
| noun_rate | 0.017 | 0.452 | 0.823 |
| adverb_rate | 0.016 | 0.463 | 0.823 |
| F1bandwidth_sma3nz_amean | 0.014 | 0.492 | 0.827 |
| F0semitoneFrom27_5Hz_sma3nz_amean | 0.012 | 0.535 | 0.827 |
| verb_phrase_with_vbg_rate | 0.011 | 0.537 | 0.823 |
| StddevVoicedSegmentLengthSec | 0.011 | 0.543 | 0.689 |
| determiner_rate | 0.01 | 0.551 | 0.823 |
| StddevUnvoicedSegmentLength | 0.011 | 0.551 | 0.689 |
| F3frequency_sma3nz_amean | 0.011 | 0.556 | 0.827 |
| inflected_verb_rate | 0.01 | 0.57 | 0.823 |
| word_frequency_min | 0.01 | 0.571 | 0.965 |
| word_frequency_max | 0.009 | 0.571 | 0.965 |
| logRelF0_H1_H2_sma3nz_stddevNorm | 0.008 | 0.591 | 0.827 |
| F1frequency_sma3nz_stddevNorm | 0.007 | 0.64 | 0.827 |
| F2bandwidth_sma3nz_stddevNorm | 0.007 | 0.644 | 0.827 |
| logRelF0_H1_A3_sma3nz_stddevNorm | 0.006 | 0.645 | 0.827 |
| F1bandwidth_sma3nz_stddevNorm | 0.006 | 0.656 | 0.827 |
| pronoun_rate | 0.005 | 0.689 | 0.896 |
| F2frequency_sma3nz_stddevNorm | 0.005 | 0.689 | 0.827 |
| mean_frazier_depth | 0.003 | 0.752 | 0.965 |
| VoicedSegmentsPerSec | 0.003 | 0.769 | 0.769 |
| number_of_pauses | 0.002 | 0.784 | 0.98 |
| honore_stat | 0.002 | 0.788 | 0.965 |
| pause_durations_sum | 0.002 | 0.824 | 0.98 |
| F2frequency_sma3nz_amean | 0.001 | 0.845 | 0.914 |
| logRelF0_H1_H2_sma3nz_amean | 0.001 | 0.845 | 0.914 |
| F1frequency_sma3nz_amean | 0.0 | 0.909 | 0.914 |
| adjective_rate | 0.0 | 0.911 | 0.959 |
| F3bandwidth_sma3nz_stddevNorm | 0.0 | 0.914 | 0.914 |
| loudness_sma3_stddevNorm | 0.0 | 0.921 | 0.921 |
| pause_durations_std | 0.0 | 0.941 | 0.98 |
| verb_phrase_with_aux_rate | 0.0 | 0.942 | 0.959 |
| word_frequency_mean | 0.0 | 0.947 | 0.965 |
| number_deictic_terms | 0.0 | 0.959 | 0.959 |
| brunets_index | 0.0 | 0.965 | 0.965 |
| duration | 0.0 | 0.98 | 0.98 |
| neutral_sentence_ratio | 0.0 | 0.99 | 0.99 |

Interaction effects between male and female participants for each speech feature

Note. The coefficient of determination (*R^2^*) quantifies the proportion of variance in the dependent variable explained by the independent variables in the regression model, including the interaction terms. *p*=unadjusted *p* value; Adj. *p*=*p* value adjusted for multiple hypothesis testing according to Benjamini-Hochberg procedure.
